# Financial Resources and Medicaid Enrollment After Dementia Onset: Findings From the Health and Retirement Study

**DOI:** 10.1093/geroni/igaf122.1443

**Published:** 2025-12-31

**Authors:** Jennifer Reckrey, Karen Shen

**Affiliations:** NYU Grossman School of Medicine, New York, New York, United States; Johns Hopkins University, Baltimore, Maryland, United States

## Abstract

People living with dementia (PLWD) frequently require high levels of paid home care for extended periods of time so that they can remain living at home. While Medicaid may cover some care costs for those who qualify, PLWD throughout the income spectrum experience significant financial burden as they pay out-of-pocket for home care. In this study, we use longitudinal data from the Health and Retirement Study between 2006-2018 to: 1) characterize financial resources available to fund home care among people with new-onset dementia, and 2) identify the proportion who go on to receive Medicaid following dementia onset. We operationalize financial resources as the ability to pay for full-time home care (40 hours per week, $16.00/ hour) over time considering self-reported income and annuitized wealth. We identified 6,228 unique PLWD with new-onset dementia between 2006-2018, 24% of whom had Medicaid at dementia onset. Among those without Medicaid, 51% had financial resources such that they could afford full-time home care for 6 months. 43%, 35%, 24% could afford full-time home care for 1, 2, and 5 years respectively. Two years after dementia onset, 7% now received Medicaid. Four years later, that proportion had grown to 14%. Few PLWD have the financial resources to support long-term paid care in the community. While Medicaid enrollment is one pathway to receive care, further work is needed to identify and characterize innovative policy and programmatic solutions to ensure that all PLWD—regardless of financial resources—can have their care needs in the community.

